# Market Confidence Predicts Stock Price: Beyond Supply and Demand

**DOI:** 10.1371/journal.pone.0158742

**Published:** 2016-07-08

**Authors:** Xiao-Qian Sun, Hua-Wei Shen, Xue-Qi Cheng, Yuqing Zhang

**Affiliations:** 1 University of Chinese Academy of Sciences, Beijing, China; 2 CAS Key Laboratory of Network Data Science & Technology, Institute of Computing Technology, Chinese Academy of Sciences, Beijing, China; East China University of Science and Technology, CHINA

## Abstract

Stock price prediction is an important and challenging problem in stock market analysis. Existing prediction methods either exploit autocorrelation of stock price and its correlation with the supply and demand of stock, or explore predictive indictors exogenous to stock market. In this paper, using transaction record of stocks with identifier of traders, we introduce an index to characterize market confidence, i.e., the ratio of the number of traders who is active in two successive trading days to the number of active traders in a certain trading day. Strong Granger causality is found between the index of market confidence and stock price. We further predict stock price by incorporating the index of market confidence into a neural network based on time series of stock price. Experimental results on 50 stocks in two Chinese Stock Exchanges demonstrate that the accuracy of stock price prediction is significantly improved by the inclusion of the market confidence index. This study sheds light on using *cross-day* trading behavior to characterize market confidence and to predict stock price.

## Introduction

With the increasing availability of huge databases for financial systems, financial study becomes a hot research topic. Scientists attempted to understand the statistical mechanics of financial systems, e.g., analyzing long-term trend and fluctuation of stock indices [1–3], modeling critical phenomenon in stock market [4–6], and anomaly detection of trading behavior [7–9]. Among them, one important research problem is stock price prediction, which has received a great deal of attention in financial studies. One central theoretical proposition about price dynamics is the efficient market hypothesis, asserting that prices effectively reflect all the relevant information available to market traders [10–14]. According to efficient market hypothesis, price dynamics is a kind of random walk and thus cannot be predicted with the prediction accuracy higher than random guess [15]. However, driven by profit or other incentives, many investors, stock analysts and even scientists devoted efforts to predict stock price.

Existing methods for stock price prediction generally fall into three main paradigms. The first kind of methods predicts stock price by exploiting autocorrelation of price dynamics [16–18]. Sun et al. proposed to combine historical price and types of trades to predict stock price [19]. Notwithstanding the existence of long-range autocorrelation in stock price, the prediction capability of these methods is limited by the fat-tailed distribution of price volatility and price return. The second kind of methods is based on trading behavior of investors, e.g., trading volume and the number of trades, with the assumption that stock price reflects the collective judgment of investors to the fundamental value of stock and thus stock price is determined by the supply and demand of stock if without exogenous interference [20, 21]. However, investors’ trading behavior is not always rational behavior, and investors could adopt quite different trading strategy. Consequently, the imbalance between supply and demand is not an effective indicator of stock price movement. Another kind of methods makes prediction of stock price by extracting some predictive indicators from exogenous sources [22–26]. Bollen et al. proposed to use the collective mood states derived from Twitter to predict the Dow Jones Industrial Average with the accuracy up to 87.6 percent [22]. Zheludev et al. used sentiment of the message in social media to predict financial markets [23]. Preis et al. identified online precursors for stock market movement, leveraging search volume data provided by Google Trends [24]. Bordino et al. found that queries pertaining to a particular stock in Web search and the daily exchange volume have a time-lagged correlation [26]. However, these methods are effective only when these exogenous sources contain potential indictors for stock price, not guaranteed in most cases.

Stock price is actually the outcome of a game among investors. For a particular stock, each investor has his/her own estimation to the fundamental value of the stock. Investors submit ask price or bid price to stock exchange, with these prices reflecting their estimation to the value of stock. Next, stock exchange executes ask orders and bid orders according to predefined rules, and stock price is a result of the execution of orders. In this process, investors’ estimation to the value of stock is the determinant of stock price. The estimated value of a stock is a full reflection of investors’ judgment according to all relevant information they could get about the stock. To buy or to sell a certain stock reflects a trader’s expectation of the movement trend of price. The decision of an individual investor may be based on incomplete information, but the collective behavior of all investors could remedy the lack of information and finally determines the price of a stock. Therefore, stock price dynamics reflects investors’ estimation to the trend of the value of stock. Investors’ confidence to their estimation offers us a natural predictive indicator for stock price.

In this paper, we study the problem of stock price prediction from the perspective of market confidence. Different from existing methods that attempt to capture market confidence by their behavior in contexts that are exogenous to stock market, we propose to extract market confidence directly from stock transaction records. Our study is conducted on transaction data of 50 stocks in two Chinese Stock Exchanges. The data contains the identifier of traders in each transaction, allowing us to identify how active each trader is. To characterize market confidence, we check the fraction of traders who also participate trades in the immediately previous trading day. Using Granger causality test, we find that stock price is strongly correlated with an index of market confidence, i.e., the ratio of the number of traders who is active in two successive trading days to the number of active traders in a certain trading day. By combining the market confidence index together with time series of stock price, we propose a stock price prediction model based on feed forward neural network. Results demonstrate that the accuracy of stock price prediction is significantly improved by the inclusion of the market confidence index. Furthermore, we investigate four types of trading patterns for traders that participate trades in two successive trading days: buy-buy, buy-sell, sell-buy, and sell-sell. We find a negative correlation between trading pattern buy-buy and price return, and sell-sell is the most relevant pattern with stock price. For manipulated stock, buy-sell is the most relevant trading pattern to stock price.

## Results

### Supply and demand of stock

Price is determined by supply and demand. In stock market, supply and demand is reflected by the trading activity of investors, i.e., sell and buy. Stock price is correlated with the number of sellers and the number of buyers [7]. Here we use Granger causality test to verify whether supply and demand of stock could be used to predict the change of stock price (See Methods). Specifically, for each trading day *t*, we consider three quantities, i.e., the number of sellers $N_{t}^{s}$, the number of buyers $N_{t}^{b}$, and the ratio of sellers $r_{t}^{s}=N_{t}^{s}/(N_{t}^{b}+N_{t}^{s})$. For each quantity, we test whether its value in the past *n* (1 ≤ *n* ≤ 7) days are useful at predicting stock price in the current day.

**Fig 1:**
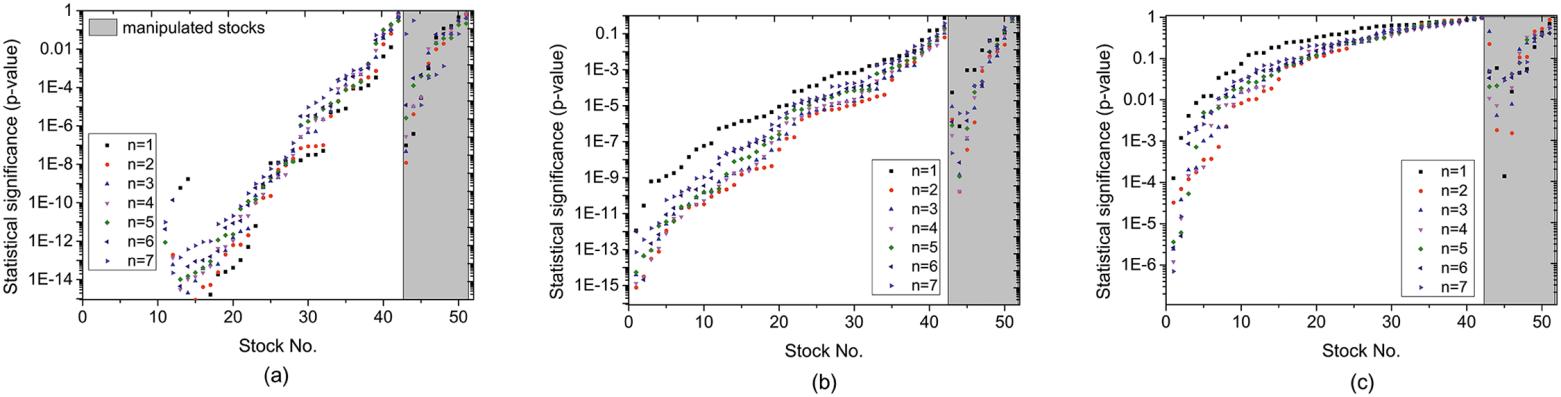
Statistical significance of bivariate Granger causality correlation between the change of stock price and (a) the ratio of sellers, (b) the number of sellers, and (c) the number of buyers. For clarity, we separately show the results of manipulated stocks and non-manipulated stocks. Stocks are ranked according to the statistical significance.

Fig 1 shows the results of Granger causality test. First, compared with the ratio of sellers and the number of sellers, the number of buyers is much insignificant at predicting the change of stock price. Only 8 stocks exhibits significant Granger causality with *p*-value < 0.01 between the time series of stock price and the time series of the number of buyers, while significant Granger causality is observed in 39 stocks when the number of sellers is considered. Second, Granger causality between the change of stock price and the supply and demand of stock is generally less significant for manipulated stocks than non-manipulated stocks. Finally, we can see that the significance of Granger causality varies remarkably from stock to stock, indicating that the supply and demand of stock is not a robust indicator for stock price prediction.

### Market confidence

The unreliability of supply and demand at predicting stock price motivates us to find alternative indicators for stock price prediction from investors’ trading behavior. From this perspective, our data possesses a unique advantage, i.e., it contains trader identifier in each executed transaction. With trader identifier, we could investigate the trading behavior of the same trader across trading days. Based on cross-day trading behavior, we propose an index to characterize market confidence of investors. Specifically, for a given trading day *t* with $N_{t}=N_{t}^{s}+N_{t}^{b}$ traders, we check whether these traders are also active in the previous trading day. We denote with $N_{t}^{a}$ the number of traders who are active in both trading day *t* − 1 and trading day *t*. We define a *market confidence index* as

$$\begin{array}{c} r_{t}^{a}=\frac{N_{t}^{a}}{N_{t}}. \end{array}$$ (1)

Market confidence index characterizes the fraction of traders who are active traders in two successive trading days. Generally speaking, traders that prefer swing trading make profit by trading frequently. Thus, the number of these traders is potentially correlated with the change of stock price.

### Stock price prediction

We now validate whether the proposed market confidence index is effective at predicting the change of stock price. Given a stock, for each trading day *t*, we extract the ratio of sellers $r_{t}^{s}$, the market confidence index $r_{t}^{a}$, and the change of stock price Δ*p*_*t*_ (See Methods), resulting in three times series. We predict the change of stock price by deploying a three-layered feed forward neural network. To distinguish predictive power of market confidence index, we consider two groups of inputs in our neural network: (1) the change of stock price in the *n* trading days before trading day *t* and the ratio of sellers in these days; (2) with the market confidence index included besides the above two inputs. These two groups of inputs are formally written as

$$\begin{array}{c} I_{1}=\{{\left\{\Delta p_{i}\right\}}_{i=t-1}^{t-n},{\left\{r_{i}^{s}\right\}}_{i=t-1}^{t-n}\}, \end{array}$$ (2)

$$\begin{array}{c} I_{2}=\{{\left\{\Delta p_{i}\right\}}_{i=t-1}^{t-n},{\left\{r_{i}^{s}\right\}}_{i=t-1}^{t-n},{\left\{r_{i}^{a}\right\}}_{i=t-1}^{t-n}\}. \end{array}$$ (3)

**Fig 2:**
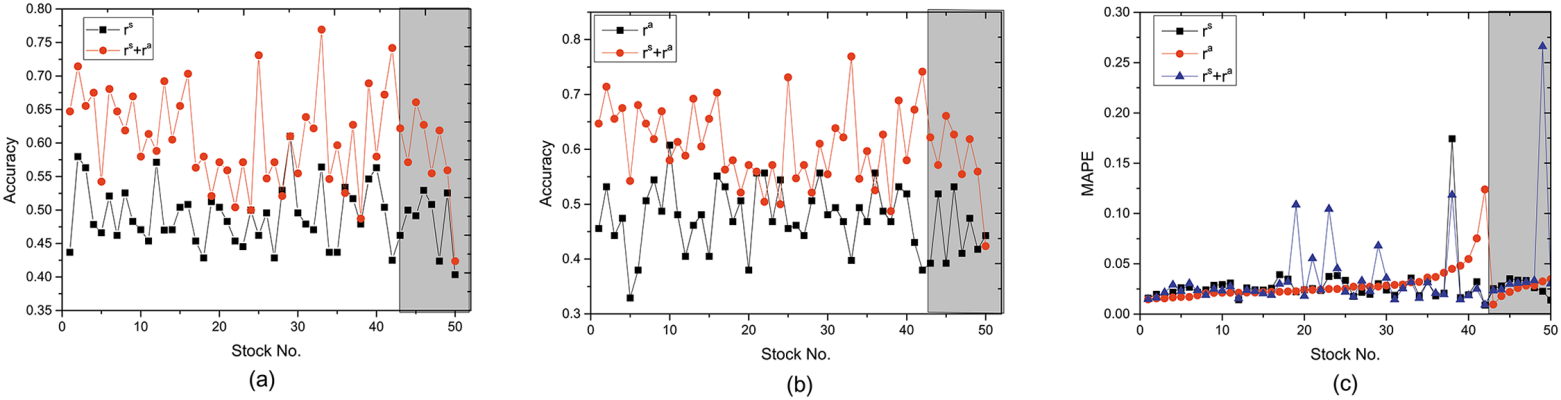
Accuracy and MAPE of stock price prediction.

**Fig 3:**
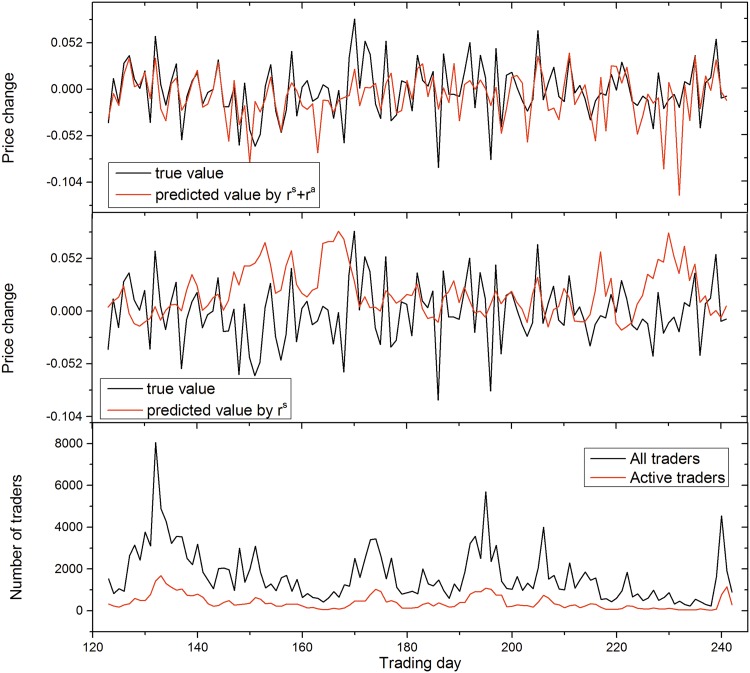
One example to illustrate the predicted results of the methods with or without market confidence index.

We use two metrics, i.e., accuracy and MAPE (see Methods), to evaluate the performance of stock price prediction. Results are shown in Fig 2 with lag *n* = 3. When incorporating market confidence index, the prediction accuracy significantly outperforms the method that only uses the ratio of sellers and historical stock price, increasing from 60% to 76.9%. Meanwhile, the MAPE is less than 0.12, with the error being smaller than the baseline in most stocks. However, when only using the market confidence index for stock price prediction, the prediction performance is not remarkable. To offer some intuition about the prediction performance, we use one example to show the predicted change of stock price and the real change of stock price (Fig 3). We can see that the index of market confidence is more stable than the ratio of sellers and it captures the long-term trend of stock price, partly explaining why the inclusion of market confidence is useful to predict the change of stock price.

We also perform the method on manipulated stock data set. We can see that MAPE is below 5% for eight manipulated stocks. The accuracy of prediction is below 66%. For manipulated stocks, the prediction accuracy is lower. One possible reason is that the stock price is manipulated by colluded traders and becomes less predictable using supply-demand relationship. Manipulation detection is a research topic with high relevance to stock market analysis. This topic is out of the scope of this paper. Here we show the difference of manipulated and non-manipulated stocks by analyzing different types of trading relationship.

## Distinguishing non-manipulated stocks from manipulated stocks

**Fig 4:**
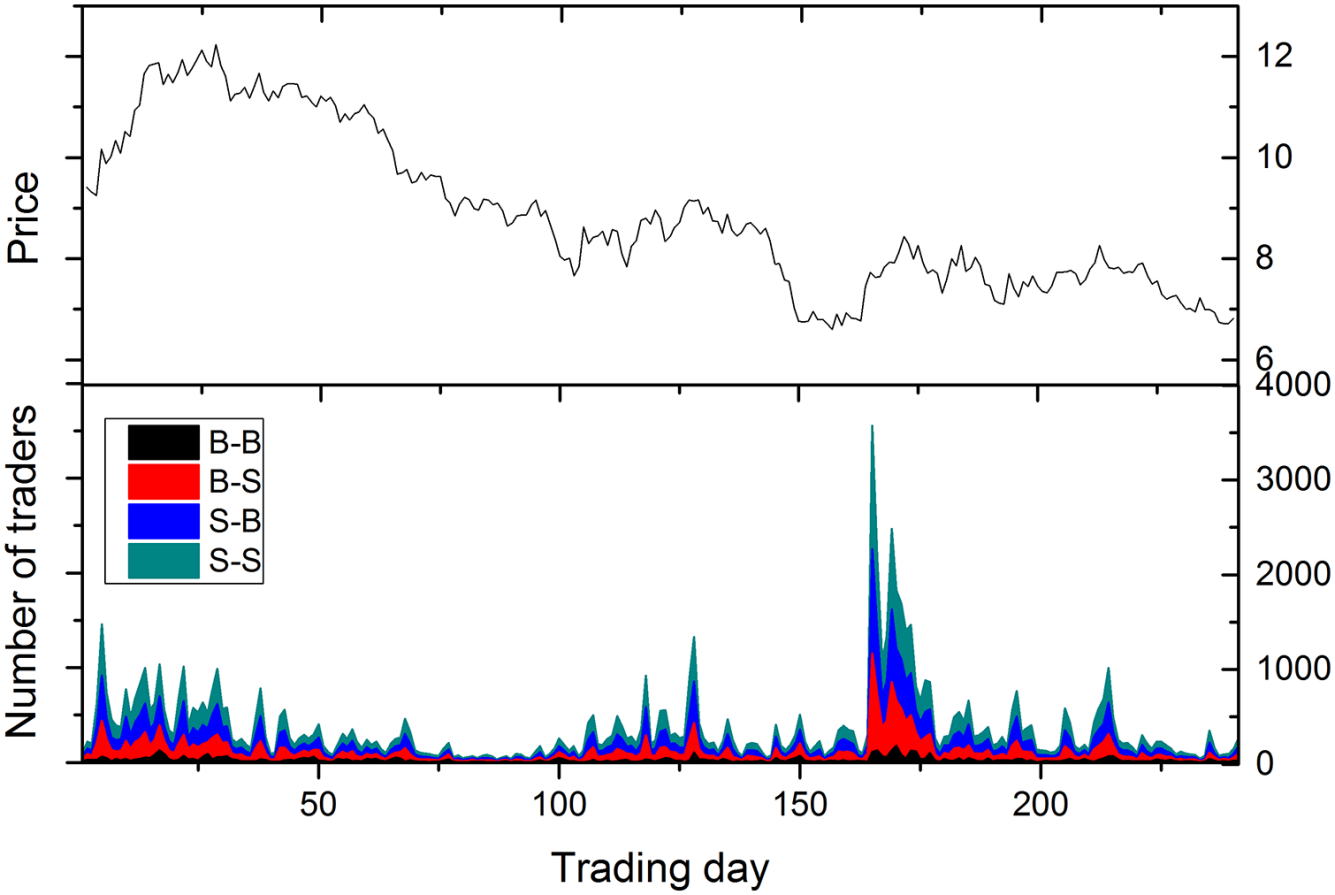
Evolution of number of active traders with the trading days for one randomly chosen stock. The price is presented in the upper panel and the four kinds of trading patterns in two successive days are shown in the bottom panel.

In the previous section, we see that the proposed method for stock price prediction exhibits different performance at manipulated stocks and non-manipulated stocks. To clarify what matters in the proposed prediction method, we classify active traders (i.e., the traders who participate trades in two successive trading days) into four categories according to their trading patterns in two successive trading days, being B-B, B-S, S-B, and S-S respectively. The first letter denotes sell or buy in the first day and the second letter denotes sell or buy in the second day. We denote the number of traders in each category as *N*_*B*−*B*_, *N*_*B*−*S*_, *N*_*S*−*B*_, and *N*_*S*−*S*_. In this way, we analyze the correlation between these four trading patterns and stock price. Fig 4 illustrates the change of the daily price with the number of active traders in each category.

**Fig 5:**
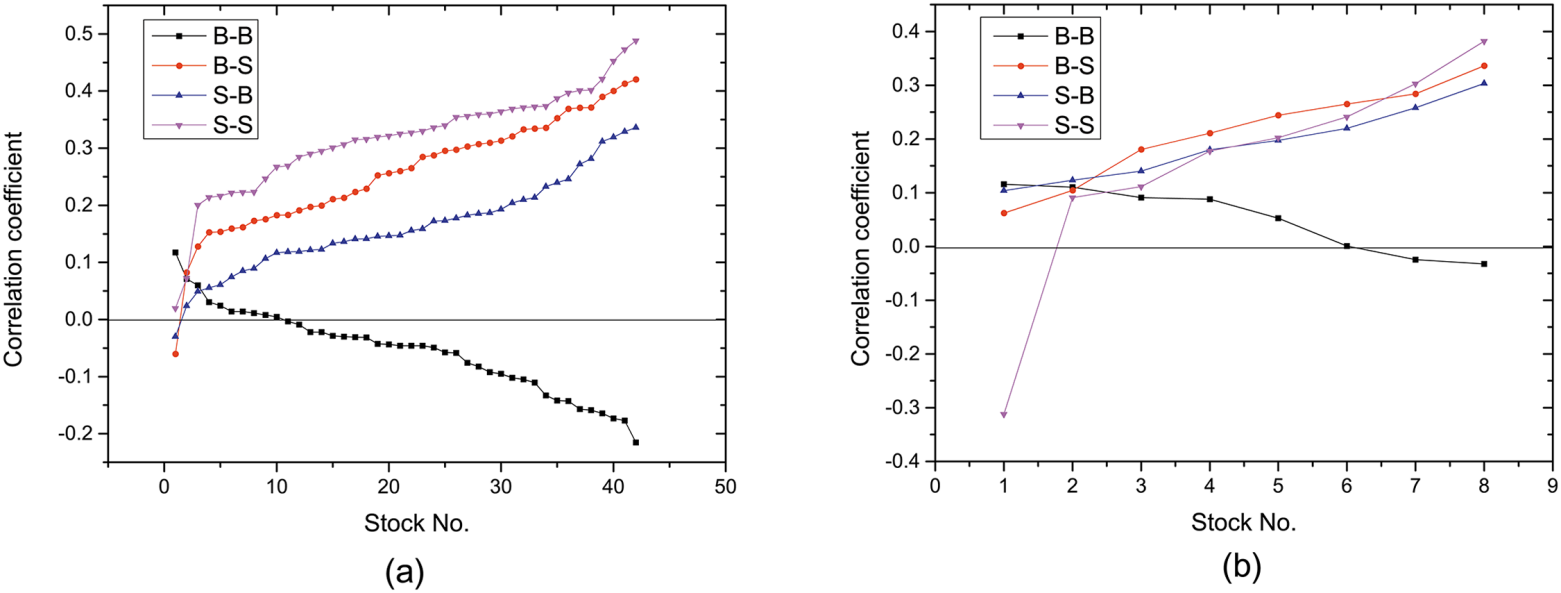
Correlation coefficient between the change of stock price and the number of traders in four kinds of trading pattern on (a) 42 non-manipulated stocks and (b) 8 manipulated stocks. Stocks are ranked in terms of correlation coefficient.

Active traders provide critical indictors for understanding trading behavior. As an illustration, we now show that the distribution of active traders over four categories could differentiate manipulated stocks from non-manipulated stocks. Fig 5 illustrates the correlation coefficient between the number of traders in each kind of trading pattern and the change of stock price. Remarkable differences are observed in the trading pattern B-B. For non-manipulated stocks, there is a negative correlation between *N*_*B*−*B*_ and the change of stock price for most stocks. Compared with non-manipulated stocks, manipulated stocks behave differently. We find that the correlation between *N*_*B*−*B*_ and the change of stock price is not significant. This has two implications. First, price determines traders’ behavior for non-manipulated stocks. If some people buy in the first day and still buy in the second day, the stock price falls. For manipulated stocks, this phenomenon diminishes. Second, compared to other trading pattern, *S* − *S* is the most relevant with the change of stock price. In contrast, the pattern *B* − *S* is positively correlated with stock price for manipulated stocks. This means that there are more short-term investment in manipulated stocks. This phenomenon is attributed to some malpractices involving a group of traders trading with large and frequent trades. Manipulators trade frequently to artificially increase the price and volume of a stock for the purpose of attracting other investors to buy the stock.

## Discussions

We investigated the dynamic behavior of traders in stock markets. Our study is based on transaction data, i.e., the executed transaction generated by electronic trading system in stock exchange. This kind of data provides us an effective way to grasp the trading relationship among investors and provides us a potential way to learn the trading behavior of investors. Based on transaction data, we consider the supply-demand relationship for each stock. We study whether trading behavior could predict the change of stock price.

Using transaction record of stocks with identifier of traders, we introduce an index to characterize investors’ confidence to the estimated value of stock. Strong Granger causality is found between stock price and the index of market confidence, i.e., the ratio of the number of traders who is active in two successive trading days to the number of active traders in a certain trading day. We further deployed a feed forward neural network to predict stock price, with the input being historical stock price, trading activity, and market confidence. Results showed that the inclusion of market confidence could significantly improve the prediction accuracy of stock price. Then we refine the active traders’ behavior into four trading pattern: buy-buy, buy-sell, sell-buy, and sell-sell. We find a negative correlation between trading pattern buy-buy and the change of stock price, and sell-sell is the most relevant pattern to the change of stock price. For the manipulated stock, buy-sell is most relevant to the change of stock price. This phenomenon means manipulators affect stock price by frequent short-term trade shares.

## Methods

### Data

The data used in this paper are transaction data of stocks listed on Shanghai Stock Exchange and Shenzhen Stock Exchange in 2004. This data is also used in our previous studies [19]. Transaction data record all executed orders. In total, the data consist of 50 stocks with 12,951,798 transaction entries, involving 3,636,876 unique trader accounts. Each entry records the date and time of transaction, a unique transaction identifier, the buyer, the seller, the volume and the price. Among all these 50 stocks, eight stocks had been manipulated by some investors via trade-based manipulation, as revealed by China Securities Regulatory Commission (CSRC). In addition, among the eight manipulated stocks, the manipulation period of four stocks persists through the whole year of 2004. For the other four manipulated stocks, the manipulation period covered by our data is from Jan. 2004 to Sep. 2004, indicating that these stocks are in the late manipulation period.

### Granger causality test

Following the method used in our previous works [19], we use granger causality test to verify whether the proposed market confidence index is promising at stock price prediction. Granger causality test is a statistical hypothesis test for determining whether one time series is useful in forecasting the other one. According to Granger causality test, if a signal *X* exhibits a statistically significant correlation with a signal *Y*, then the past values of *X* should contain information that helps predict *Y* better than only leveraging the information contained in past values of *Y*.

In this paper, we use Granger causality test to judge whether trading activity and market confidence are useful at forecasting the change of stock price. The change of stock price at day *t* is defined as

$$\begin{array}{c} \Delta p_{t}=\ln p_{t}-\ln p_{t-1}, \end{array}$$ (4)

where *p*_*t*_ is the opening price at day *t*. Trading activity is characterized by the ratio of sellers in the traders at each trading day. For each trading day, market confidence is characterized by the fraction of traders who sell or buy stocks in previous trading day. The null-hypothesis for our Granger causality test is that trading activity or market confidence Granger-causes the change of stock price. With *X* denoting the time series of trading activity or market confidence, the test is conducted by comparing the following two regression models

$$\begin{array}{c} \Delta p_{t}=\alpha +\sum_{i=1}^{n}\beta_{i}\Delta p_{t-i}+\xi_{t}, \end{array}$$ (5)

$$\begin{array}{c} \Delta p_{t}=\alpha +\sum_{i=1}^{n}\beta_{i}\Delta p_{t-i}+\sum_{i=1}^{n}\gamma_{i}X_{t-i}+\xi_{t}, \end{array}$$ (6)

where *α*, *β*, *γ* are model parameters, and *ξ* denotes prediction error. In the first linear regression model, *n* lagged values of the change Δ*p* of stock price are used to predict its future value. In the second linear regression model, both the lagged values of Δ*p* and *X* are employed for prediction. The Granger causality analysis is conducted on all the 50 stocks in our data. Before Granger causality test, we use Augmented Dickey-Fuller (ADF) test to test our time series data, and find that the non-stationarity hypothesis is rejected at the significant level of 0.01 for all the stocks.

### Stock price prediction using neural network

Granger causality test could provide some insight about which trading activity is potential at predicting stock price. However, Granger causality test is based on linear regression model and thus cannot uncover the relevant factors which are non-linearly predictive for stock price. To address this problem, we develop a three-layered feed forward neural network model which is non-linear model and could fully exploit the potential prediction power of its input.

To train the neural network, we divide all the data into two equal-sized parts: the training set and the test set. For the stocks in training set, the future stock price is used to train the neural network. For the stocks in test set, only the past time series of stock price, trading activity, and market confidence are known. To assess the role of trading activity and market confidence, we compare the performance of neural networks with two different sets of inputs: (1) the time series of stock price and the time series of trading activity; (2) the time series of stock price, the time series of trading activity, and the time series of market confidence. The output of neural networks is the change of stock price.

The effectiveness of prediction method is measured in terms of the Mean Absolute Percentage Error (MAPE) and the accuracy at predicting the rise or fall of stock price. MAPE is a measure to evaluate the accuracy of the predicted time series relative to the real time series. Denoting with *A*_*t*_ the real change of stock price and *F*_*t*_ the predicted change of stock price, MAPE is defined as:

$$\begin{array}{c} MAPE=\frac{1}{n}\sum_{t=1}^{n}|\frac{A_{t}-F_{t}}{A_{t}}|. \end{array}$$ (7)

For accuracy, we just evaluate whether the predicted trend (i.e., rise or fall) of stock price is consistent with the real trend of stock price.
